# Impact of diastolic pulmonary gradient and pulmonary artery pulse index on outcomes in heart transplant patients—Results from the Eurotransplant database

**DOI:** 10.3389/fcvm.2022.1036547

**Published:** 2022-12-16

**Authors:** Tobias Wagner, Christina Magnussen, Alexander Bernhardt, Jacqueline M. Smits, Katrin Steinbach, Hermann Reichenspurner, Paulus Kirchhof, Hanno Grahn

**Affiliations:** ^1^Department of Cardiology, University Heart and Vascular Center Hamburg, Hamburg, Germany; ^2^German Center for Cardiovascular Research (DZHK), Partner Site Hamburg/Kiel/Luebeck, Hamburg, Germany; ^3^Department of Cardiovascular Surgery, University Heart and Vascular Center Hamburg, Hamburg, Germany; ^4^Eurotransplant International Foundation, Leiden, Netherlands; ^5^Department of Cardiology, University Medical Centre, Johannes Gutenberg University Mainz, Mainz, Germany; ^6^Institute of Cardiovascular Sciences, University of Birmingham, Birmingham, United Kingdom

**Keywords:** orthotopic heart transplantation, pulmonary hypertension, diastolic pulmonary vascular pressure gradient, transpulmonary pressure gradient, pulmonary pulsatility index

## Abstract

**Background:**

Predicting complications associated with pulmonary hypertension (PH) after cardiac transplantation is an important factor when considering cardiac transplantation. The transpulmonary gradient (TPG) is recommended to quantify PH in transplant candidates. Nonetheless, PH remains a common driver of mortality. The diastolic pressure gradient (DPG) and pulmonary vascular resistance (PVR) can differentiate post- from combined pre- and post-capillary PH and may improve estimation of PH-associated risks. We used a large European cohort of transplant candidates to assess whether the pulmonary pulsatility index (PAPi), improves prediction of graft failure and mortality compared to DPG and PVR.

**Methods:**

Out of all patients undergoing heart transplantation between 2009 and 2019 in Eurotransplant member states (*n* = 10,465), we analyzed the impact of PH (mPAP > 25 mmHg) and right heart catheter hemodynamic data on graft failure and mortality within 1–5 years.

**Results:**

In 1,407 heart transplant patients with PH (79% male, median age 54 years, IQR 39–69 years), the median PVR was 2.5 WU (IQR 1.6 WU) with a median mPAP (pulmonary arterial pressure) of 32 mmHg (IQR 9 mmHg). Patients with low (< 3 mmHg) DPG had a better 5 year survival than those with higher DPG (log rank *p* = 0.023). TPG, mPAP, PAPi, and PVR did not improve prediction of survival. Low PAPi (*OR* = 2.24, *p* < 0.001) and high PVR (*OR* = 2.12, *p* = 0.005) were associated with graft failure.

**Conclusion:**

PAPI and PVR are associated with graft failure in patients with PH undergoing cardiac transplantation. DPG is associated with survival in this cohort.

## Introduction

Cardiac transplantation is gold standard of care for patients with advanced heart failure. One of the main drivers of morbidity and mortality after cardiac transplantation is right heart failure, often due to pre-existing pulmonary hypertension (PH). PH is often present in patients awaiting cardiac transplant. PH can be a reversible consequence of left heart disease or a sign of irreversible defects in the pulmonary circulation. Differentiating between these etiologies can alter effects on the risk of post-transplant right heart failure (1).

Right heart catheterization (RHC) is commonly used to assess candidates for cardiac transplantation as it allows quantification of cardiac output and calculation of cardiac index. RHC also provides comprehensive right heart and pulmonary hemodynamic information. The (2) pulmonary arterial wedge pressure (PAWP), the derived transpulmonary gradient (TPG) and the calculated pulmonary vascular resistance (PVR) are currently used to differentiate pre- from post-capillary hypertension in candidates for cardiac transplantation (1).

Unfortunately, both TPG and PVR can be increased in patients with cardiac failure and subsequently increased pulmonary venous pressure (3). Therefore, TPG and PVR may over- or under-diagnose the risk of PH-associated morbidity and mortality after cardiac transplantation. The diastolic pulmonary gradient (DPG) can help to further differentiate pre- and postcapillary PH (4). Novel functional parameters like the pulmonary artery pulsatility index (PAPi) were recently proposed to better estimate the risk of right heart failure in cardiac transplant candidates (5, 6).

To quantify the value of these parameters for risk prediction after cardiac transplantation, we analyzed a large European transplant database to determine whether DGP and PAPi are associated with organ failure and death after cardiac transplantation.

## Methods

### Data source

All adult patients undergoing orthotopic heart transplantation (OHT) between 2009 to 2019 were extracted from the Eurotransplant database (*n* = 10,465). Eurotransplant is an international non-profit organization coordinating organ transplants in Austria, Belgium, Croatia, Germany, Hungary, Luxembourg, Netherlands, and Slovenia.

### Data management and study design

We examined all adult (age ≥ 18 years) OHT patients with a minimum set of pre-transplant hemodynamic data, defined as systolic pulmonary artery pressure (sPAP), diastolic pulmonary artery pressure (dPAP), mean pulmonary artery pressure (mPAP), pulmonary artery wedge pressure (PAWP), and cardiac output.

Depending on the implication for OHT and the state of urgency as well as changes in the allocation system, in a vast number of patient RHC was not mandatory of OHT listing. Patients without or with incomplete hemodynamic data excluded. To identify outliers (defined as physiologically impossible parameters), *Z*-standardization of mPAP, PAWP, dPAP, TPG and DPG was performed. All cases with at least one value >1.5^*^IQR + *3rd quartile* or < 1.5^*^IQR + *3rd quartile* were marked as an outlier and excluded from subsequent analysis. Outcomes of interest included survival at 30 days, 1 year, and 5 years as well as graft failure at any time. The cohort was divided into two groups: patients with PH (mPAP ≥25 mmHg) and patients without PH (mPAP < 25 mmHg). This threshold was recommended in the time period the patients included in this study were screened to be eligible for OHT. Recently, the revised WHO definition of PH recommend a threshold of >20 mmHg (7).

### Statistical analysis

For all variables, descriptive statistics were computed. Depending on the scale of measure, data are presented as numbers and percentages, medians and interquartile ranges, or proportions with 95% confidence intervals (*CI*). For comparison between groups, exclusively non-parametric tests were used. Continuous variables were compared by using the Mann-Whitney *U*-tests and Chi Square test was used to compare categorical variables. The predictive performances of the TPG, DPG, PVR, and PAPi were analyzed with receiver operating characteristic (ROC) curves. Total area under the ROC curve (AUC) values were considered to assess the value of measure. ROC cut points were identified by Youden's index. Kaplan-Meier curves were used to analyze survival and were compared by log-rank test. Spearman rank correlation was used to examine the correlation between variables. Dependent correlations were compared as a variant of Dunn und Clark's *z* proposed by Hittner et al. (8). Prediction models for survival and graft failure were adjusted for age, sex, listing state and underlying heart disease as possible confounders by logistic regression. For regression models, *p* < 0.05 was used as the entry criterion and *p* >0.10 as the removal criterion. Data were analyzed with IBM SPSS version 25 for Microsoft Windows. Two-tailed tests of significance were considered to be significant at a *p* < 0.05 and highly significant at *p* < 0.01.

## Results

### Patients and hemodynamic parameters

**Figure 1:**
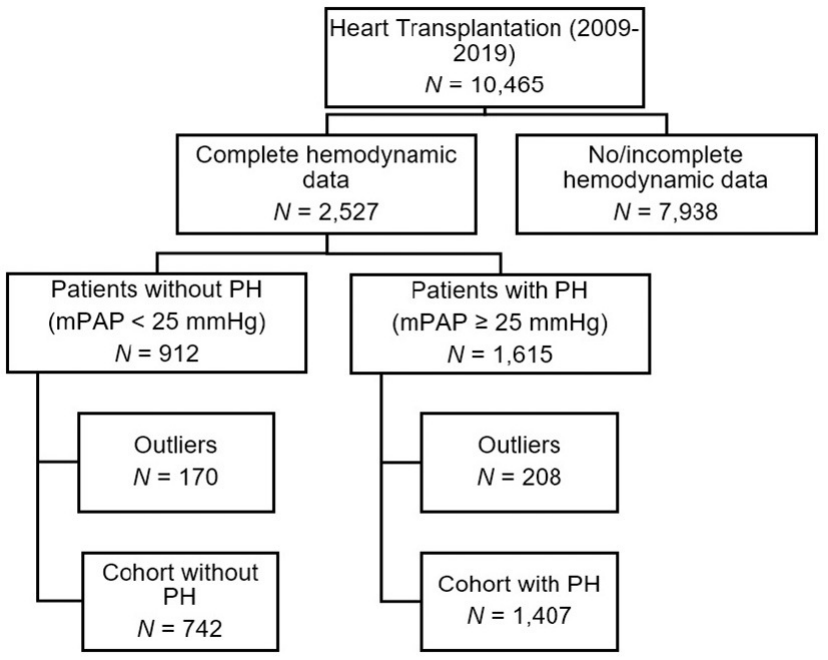
Flowchart of study population according broken down by right-heart catheterization data into pulmonary hypertension groups.

Of 10,465 cardiac transplant recipients between 2009 and 2019, 7,938 patients were excluded with incomplete hemodynamic data, and 378 patients with implausible data or outliers (Figure 1). Of the remaining cohort of 2,149 patients, 1,407 patients (65%) had pulmonary hypertension (mPAP ≥ 25 mmHg). Patients with PH were more frequently female and had higher pulmonary pressures, except DPG and PAPi, compared to the 742 transplant recipients without PH. The median time between RHC and OHT was 31 days in patients with PH, while RHC in patients without PH was performed at a median interval of 41 days before OHT (*p* < 0.001). We found a longer median follow-up time after OHT in patients with PH. In patients with PH, left ventricular assist device (LVAD) support as bridge to transplant was present half as frequently (19 vs. 9%, *p* < 0.001). No significant differences in donor characteristics between patients with and without PH were obvious (Table 1). We selected 1,407 patients with PH as the primary analysis population.

**Table 1:** Baseline characteristics.

	**No PH (mPAP < 25 mmHg) *N* = 742**	**PH (≥25 mmHg) *N* = 1,407**	***p***
**Demographics**			
Age (years)	54 *(39–69)*	54 *(39–69)*	0.190
Female sex	212 *(29)*	298 *(21)*	< 0.001
LVAD before OHT	138 *(19)*	119 *(9)*	< 0.001
ECMO or IABP before OHT	9 *(1)*	24 *(2)*	0.377
**Primary etiology of heart failure**			
Ischemic heart disease	139 *(19)*	304 *(22)*	0.117
Dilated cardiomyopathy	410 (*55*)	804 *(57)*	0.402
Congenital heart disease	20 *(3)*	31 *(2)*	0.476
Mixed/others	173 *(23)*	268 *(19)*	
**Listing state and donor characteristics**			
T	116 *(16)*	179 *(13)*	
HU	626 *(84)*	1,228 *(87)*	0.062
Age of donor (years)	44 *(24–64)*	44 *(23–65)*	0.559
BMI of donor (kg m^−2^)	25 *(20–30)*	25 *(20–29)*	0.182
Female sex of donor	306 *(41)*	569 *(41)*	0.720
**Hemodynamics**			
sPAP (mmHg)	29 *(17–41)*	48 *(32–64)*	< 0.001
mPAP (mmHg)	19 *(13–25)*	33 *(25–41)*	< 0.001
dPAP (mmHg)	14 *(7–21)*	25 *(17–33)*	< 0.001
PAWP (mmHg)	14 *(6–22)*	24 *(15–33)*	< 0.001
PVR (WU)	1.70 *(0.42–2.98)*	2.5 *(0.9–4.1)*	< 0.001
TPG (mmHg)	5 *(0–10)*	8 *(2–14)*	< 0.001
DPG (mmHg)	0 *(−5–5)*	0 *(−5–5)*	0.323
CI (L min^−1^) m^−2^)	2.00 *(1.4–2.6)*	1.81 *(1.31–2.31)*	< 0.001
CVP (mmHg)	8 *(0–17)*	13 *(3–23)*	< 0.001
PAPi	1.93 *(0–4.33)*	1.75 *(0–3.54)*	0.192
Time between RHC and OHT (days)	41 *(0–126)*	31 *(0–75)*	< 0.001
**Median follow-up time**			
Median follow-up (days)	712 (*0–1,557*)	857 (*0–1,780*)	< 0.001

Median *(IQR)* or *N (%)*, p-values are calculated by Mann-Whitney U-test or Chi Square test.

CI, cardiac index; CVP, central venous pressure; dPAP, diastolic pulmonary artery pressure; DPG, diastolic pulmonary artery pressure-to-pulmonary capillary wedge pressure gradient; HU, urgency status “high urgent”; PAPi, pulmonary artery pulsatility index; mPAP, mean pulmonary artery pressure; PH, pulmonary hypertension; PVR, pulmonary vascular resistance; T, urgency status “transplantable”; TPG, transpulmonary gradient; sPAP, systolic pulmonary artery pressure. Italic values was used to delimit interquartile range and % from median and %.

### Prognostic value of established PH parameters TPG, DPG and PVR

We investigated the ability of the established PH parameters DPG, TPG and PVR to discriminate between survivors and non-survivors in the cohort of patients with PH and found AUC values near 0.5 (Table 2). We took the conventional cut-off value of 3 mmHg for DPG and identified 361 patients (26% of patients with PH) showing a DPG above. We set cut-offs for TPG at 15 mmHg, and 3 WU for PVR.

**Table 2:** Survival in patients with a mean pulmonary artery pressure ≥25 mmHg.

**Variable**	**AUC**	***p***
**DPG**		
30-days survival	0.489	0.707
1-year survival	0.464	0.067
5-year survival	0.466	0.123
**Transpulmonary gradient**		
30-days survival	0.428	0.017
1-year survival	0.459	0.036
5-year survival	0.458	0.059
**Peripheral vascular resistance**		
30-days survival	0.417	0.051
1-year survival	0.448	0.053
5-year survival	0.415	0.118
**Pulmonary artery pulsatility index**		
30-days survival	0.500	0.995
1-year survival	0.511	0.584
5-year survival	0.481	0.408

AUC, area under the curve; DPG, diastolic pulmonary artery pressure-to-pulmonary capillary wedge pressure gradient.

**Figure 2:**
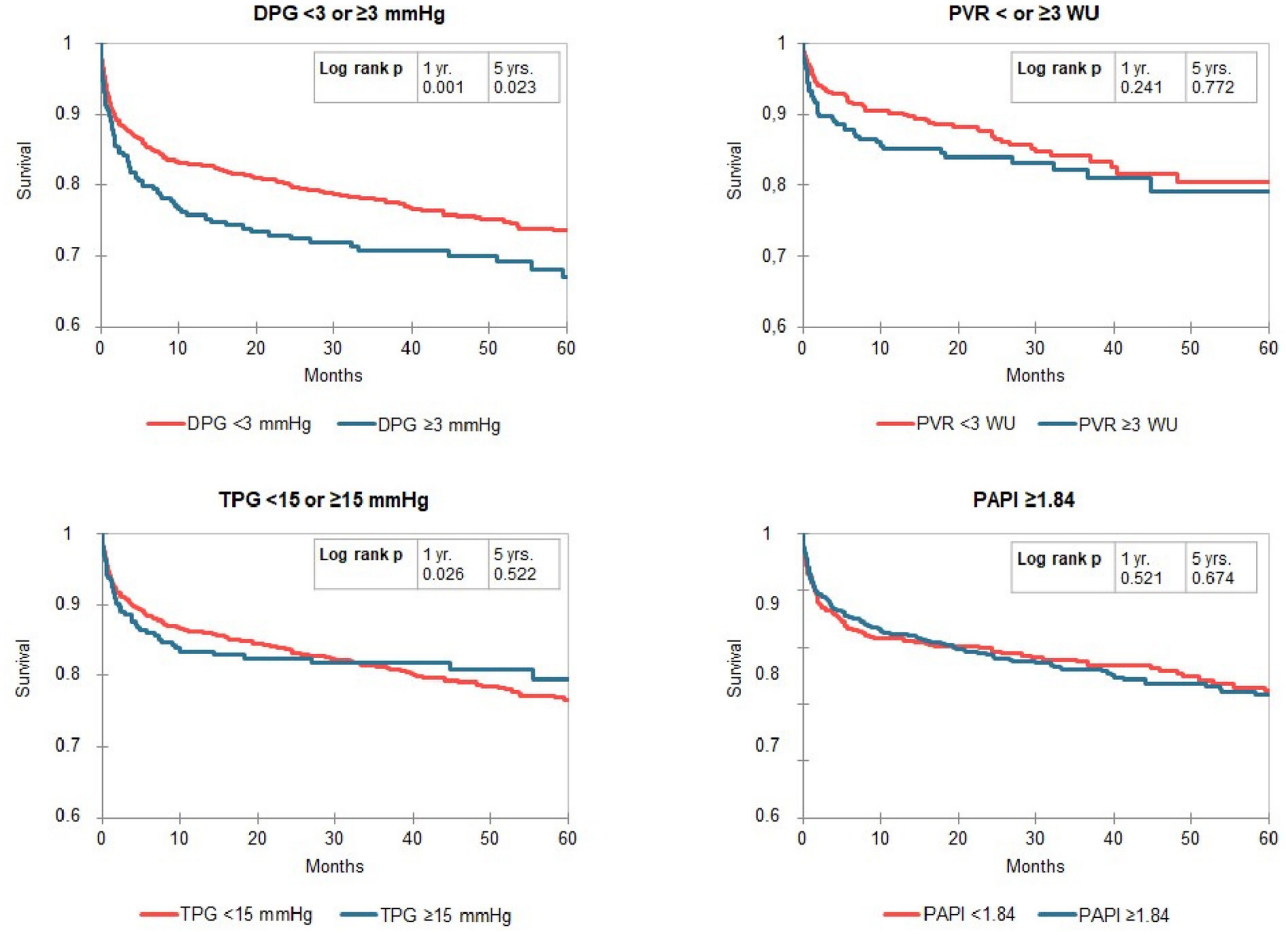
Kaplan-Meier curves for Transpulmonary pressure gradient diastolic pulmonary artery pressure-to-pulmonary capillary wedge pressure gradient (DPG), transpulmonary pressure gradient (TPG), pulmonary vascular resistance (PVR) and pulmonary pulsatility index (PAPi) dived by cut-off values in patients with pulmonary hypertension (mPAP ≥ 25 mmHg). The numbers of patients at risk are listed in Supplementary Table S4.

**Figure 3:**
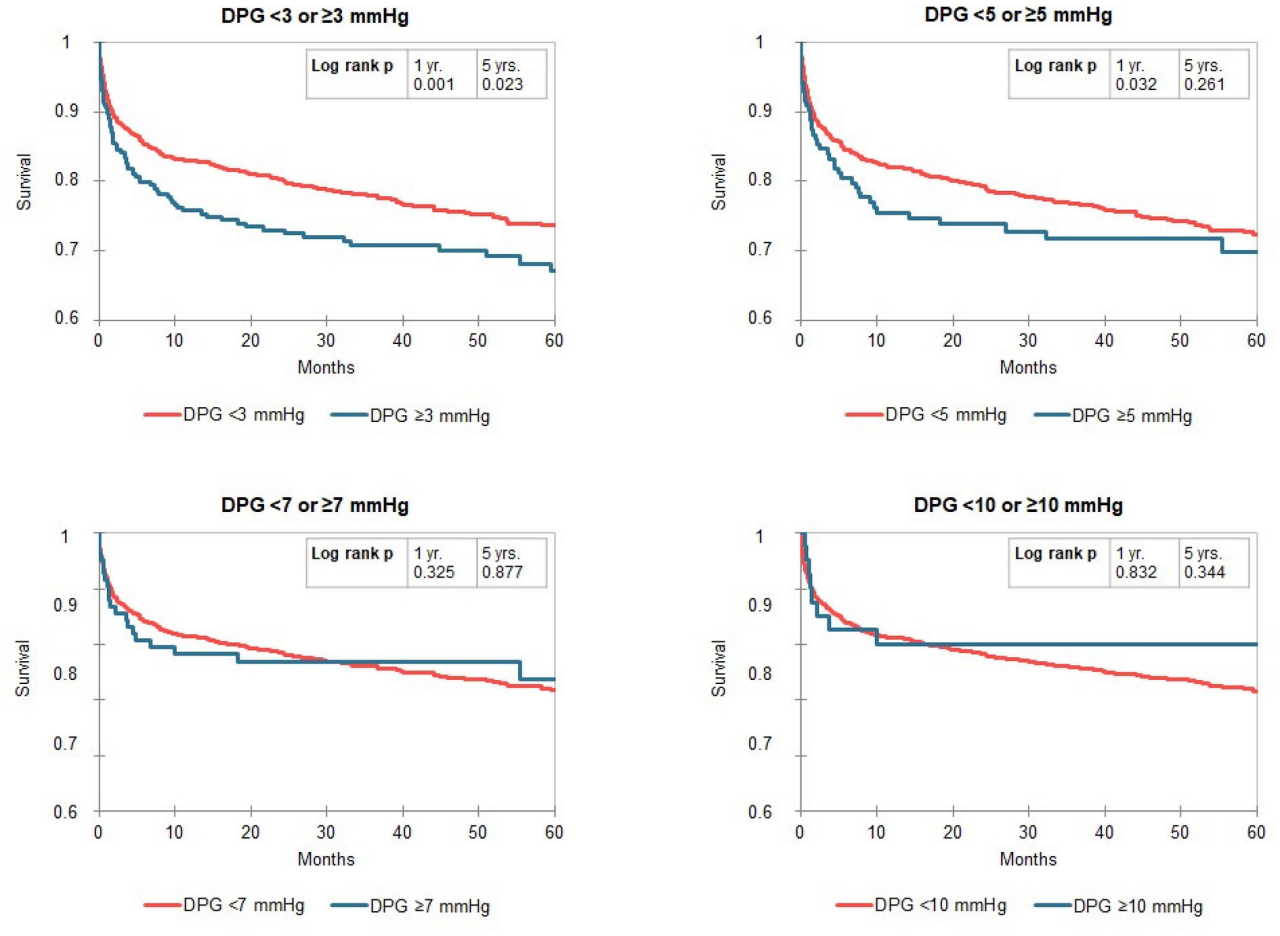
Kaplan-Meier curves for the gradient diastolic pulmonary artery pressure-to-pulmonary capillary wedge pressure gradient (DPG) split by different cut points (3, 5, 7, or 10 mmHg) in patients with pulmonary hypertension (mPAP ≥ 25 mmHg). The numbers of patients at risk are listed in Supplementary Table S4.

Kaplan-Meier curves identified a distinct better 1-year survival in patients with a DPG < 3 mmHg (log rank *p* ≤ 0.001) and a slightly better 5-year survival in the same group of patients (log rank *p* = 0.023, median survival 1,556 vs. 1,318 days). There was no difference in survival in patients with low vs. high TPG, mPAP, and PVR (Figure 2). For the above-mentioned survival analysis, we used a DPG cut point of 3 mmHg chosen a priori. Exploring three different cut points (3, 5, 7, or 10 mmHg) in this cohort of patients with PH, a cut point of 3 mmHg remains the one with best discrimination regarding 5-year survival (Figure 3). TPG and PVR were higher in the high DPG groups (Supplementary Table 2). Analyses of subgroups with PVR >3 WU or TPG >12 mmHg had similar results (Supplementary Table 3).

### Prognostic value of PAPi

Since there are few data regarding the prognostic impact of PAPi in patients with end stage heart failure, there is no established threshold for PAPi available for OHT patients. We chose 1.84 as threshold as the PAPi cutoff with the best Youden Index for 5-year survival. A PAPi ≥ 1.84 was found in 53% of patients with PH and therefore this cut-off divided the group of recipients with PH in two subgroup with nearly identic size. However, in the Kaplan-Meier curves we found no differences in compare of survival (Figure 2). Likewise, splitting the cohort by a threshold of 3.65—proposed in literature for patients with heart failure (6)—did also not reveal meaningful differences in survival, but in only 18% of recipients with PH a PAPi ≥3.65 was found (data not shown).

### Adjusted models for survival and graft failure

sPAP correlated considerably weaker with DPG (*r* = −0.11) than TPG (*r* = 0.47). This was consistent in the subgroup of patients with mPAP ≥ 25 mmHg (*r* = −0.11 vs. *r* = 0.44) and in those with PVR >3 WU (*r* = −0.15 vs. *r* = 0.43, (see Supplementary Table 3) for *p*-values between correlation coefficients). DPG was neither associated with cardiac output in the whole cohort (*r* = 0.02) nor in the subgroup of patients with mPAP ≥ 25 mmHg (*r* = 0.04).

After adjustment for age, sex, listing state and underlying heart disease as possible confounders by use of a logistic regression model, we found that patients with PH and DPG >3 mmHg had a worse survival after 1 year (odds ratio 0.63 [95% confidence interval 0.47–0.85], *p* = 0.002) and 3 years (odds ratio 0.72 [95% confidence interval 0.543–0.959], *p* = 0.024). Five years after transplantation, this difference diminished (odds ratio 0.80 [95% confidence interval 0.56–1.13], *p* = 0.181). Adding TPG as an additional parameter did not change this result (odds ratio 0.60 [95% confidence interval 0.42–0.85], *p* = 0.003 for survival after 1 year, odds ratio 0.66 [95% confidence interval 0.47–0.93], *p* = 0.016 for survival after 3 years and odds ratio 0.81 [95% confidence interval 0.54–1.22], *p* = 0.312 for survival after 5 years) indicating that this prediction is independent from TPG. In contrast, we could not depict a significant difference in survival at any point for patients with TPG > 15 mmHg, PVR > 3 WU. Further, the optimal PAPi threshold of 1.84 did not separate group with differing probability of survival.

With the aforementioned adjustment, PH and DPG > 3 mmHg (odds ratio 1.30 [95% confidence interval 0.93–1.83], *p* = 0.125) or TPG > 15 mmHg (odds ratio 0.83 [95% confidence interval 0.44–1.57], *p* = 0.571) did not increase the risk of graft failure, but PVR >3 WU (odds ratio 2.12 [95% confidence interval 1.25–3.59], *p* = 0.005) did. An optimal PAPi threshold of 2.37 discriminates two groups markedly differing in probability of graft failure (odds ratio 2.24 [95% confidence interval 1.51–3.32], *p* < 0.001, Table 3). Restricting the analysis to patients developing graft failure within the first 90 days after OHT, did not change these results (data not shown).

**Table 3:** Hemodynamic risk factors for graft failure adjusted for age, sex, listing state and underlying heart disease.

**Factor**	**OR**	**95% CI**	***p***	**OR and 95% CI**
DPG > 3 mmHg	1.30	(0.93–1.83)	0.125	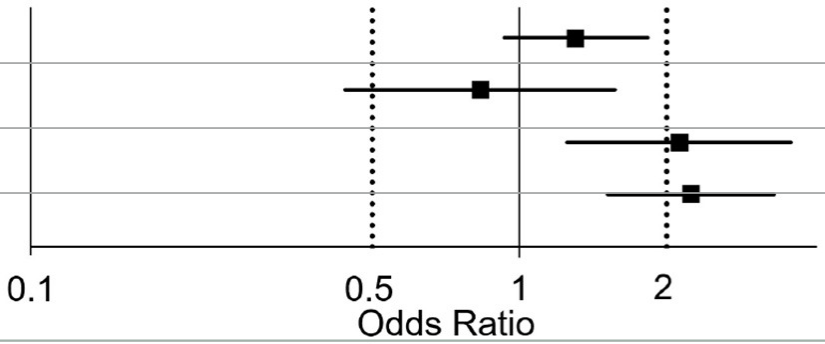
TPG > 15 mmHg	0.83	(0.44–1.57)	0.571	
PVR > 3 WU	2.12	(1.25–3.59)	0.005	
PAPi < 2.37	2.24	(1.51–3.32)	< 0.001	

DPG, diastolic pulmonary artery pressure-to-pulmonary capillary wedge pressure gradient; TPG, transpulmonary gradient; PVR, pulmonary vascular resistance; PAPi, pulmonary artery pulsatility index.

## Discussion

**Figure 4:**
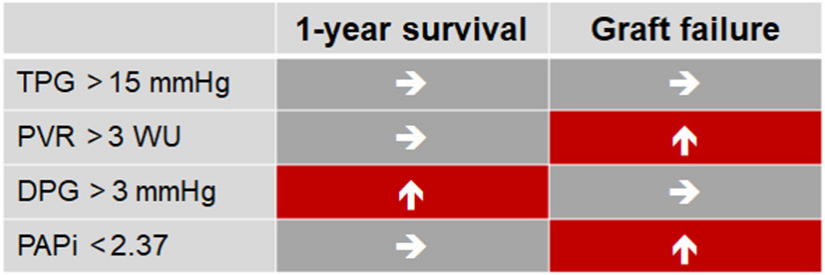
Hemodynamics risk factors of OHT in patients with PH.

This analysis in a near-complete cohort of cardiac transplant patients with pulmonary hypertension in eight European countries confirms that pulmonary hypertension is common (2/3 of patients) in patients undergoing cardiac transplantation with prior hemodynamic evaluation by RHC. Two new parameters were identified to estimate risk of death and organ failure: A low transpulmonary diastolic pressure gradient (DPG < 3 mmHg) is associated with good 5 year survival. Low PAPi and high PVR were associated with graft failure (central illustration, Figure 4). Our findings suggest that quantification of DPG and PAPi in addition to PVR can improve risk estimation in candidates for cardiac transplantation with elevated pulmonary pressure.

Frequently, TPG and PVR are used for ruling-out OHT candidates with irreversible pulmonary remodeling representing a contraindication (1). In left heart disease, TPG can be elevated not only as a result of pulmonary vascular remodeling but also due to the effects of elevated left-sided filling pressures (3). Thus, elevated TPG or PVR does not always reflect irreversible pulmonary vascular disease. TPG may misjudge pulmonary vascular disease in left heart conditions associated with an increased pulmonary venous pressure. Weak prognostic power of PVR and TPG has already been described (9). In this study, we could confirm that TPG do not predict survival or graft failure while increased PVR was associated with graft failure but did not significantly influence survival.

An increase in venous pressure lowers vascular compliance more than solely expressed by PVR (10). This phenomenon majorly increases sPAP and subsequently mPAP but without increase of dPAP. As Naeije et al. (3) describes, TPG and PVR are further highly dependent on cardiac output. Our data show that neither TPG > 15 mmHg nor elevated PVR > 3 WU was associated with worse survival.

Comparing TPG and DPG, the latter should be less affected by changes in vascular compliance induced by left heart failure and would appear as an attractive alternative to determine irreversible structural changes in pulmonary vasculature (11). According to this, DPG was independent from sPAP in our study. The evaluation of prognostic significance of the precapillary component of combined PH is complicated by conflicting definitions. While DPG was until recently only considered in the context of elevated mPAP and PAWP (12), the World Symposium on PH favored DPG for the diagnosis of combined post-capillary and pre-capillary PH as the sole discriminator in 2018, but do not longer recommend the use of DPG since 2020 (13).

The effect of elevated DPG on prognosis in PH is controversial. While Tampakakis et al. (14) found that DPG—considered continuously and at multiple cut points—did not demonstrate a significant association with survival in patients with left heart failure, Gerges et al. (15) showed that elevated DPG (≥7 vs. < 7 mmHg) was associated with worse median survival in patients with left heart disease with PH and TPG > 12 mmHg. Although Tedford et al. (16) found no impact of DPG on survival in a US cohort of nearly 6,000 patients with advanced heart failure listed for OHT, we could show that DPG > 3 mmHg in OHT candidates with PH was associated with a lower 5-year survival. When favoring DPG, one should notice that even a small error in the measured PAWP might lead to a significant change in the DPG. Therefore, verification than several parameters' suiting to each other is crucial. Further, increases in heart rate lead to an increase in DPG (17). In patients with severe heart failure, this is of interest because tachycardia might occur to compensate low cardiac output or caused by inotropic medications. For further research, especially post-transplant hemodynamic data is required to confirm the importance of DPG and to determine if and to what extent the DPG normalizes after transplantation and if persistently elevated DPG relates to worse outcomes. Goland et al. (18) have shown that failure to normalize PVR to < 3 WU after transplantation impacts long-term survival. DPG is not reported in this study.

PAPi is an indicator of right heart function and describes the function of the right ventricular unit against a given afterload. Calculated by pulmonary artery pulse and right atrial pressure its calculation is based on physiology of RV stroke volume, pulmonary arterial capacitance and right atrial pressure. PAPi has been shown to predict 6-month mortality and hospitalization in patients with advanced heart failure. However, no PAPi reference value is available in the setting OHT. Recently Guven et al. (19) found a relevant association between lower PAPi values and higher probability of kidney injury AKI severity in patients with elevated RAP. In out cohort, low PAPi doubled the risk of graft failure. Here we show an impact on graft failure favoring implementation in pre-OHT evaluation as an additional element of patient selection.

In our cohort, the median time between RHC and OHT was significantly longer in the group of patients without PH. This finding might be partly explained by a higher percentage of patients with LVAD as bridge to transplant and a slightly lower percentage of patients with high urgent listing state in this group. Further, there is less need for hemodynamic follow-up in patients without PH.

Interestingly, RHC was performed more frequent in patients with LVAD. While RHC with complete hemodynamic data was performed in 23% of OHT candidates without LVAD, in 80% of candidates with LVAD hemodynamic data was available. Possibly, some of these patients became hemodynamically eligible for transplantation only after a period of LVAD therapy.

Since LVAD has been shown to lower PVR in PH patients and improve hemodynamics for optimal post OHT outcomes (20), the lower share of PH in LVAD patients is in line with current research. Thresholds for hemodynamic parameters in OHT candidates with LVAD are under debate, but may not fundamentally differ from those in patients without LVAD. Recently, Ruan et al. (21) showed that post-LVAD PVR >3 WU negatively predicts OHT outcomes.

Several limitations of our data should be mentioned. Neither data on vaso-dynamic testing before OHT nor post-OHT hemodynamics were available in a sufficient number of cases. Probably, some patients who did not demonstrate reversibility were excluded as transplant candidates and are therefore not included in this study. Few clinical information on the patients were available, which limits data adjustment. It is likely that co-morbidities influence TPG (e.g., chronic obstructive pulmonary disease) or pulmonary artery pressure (e.g., reduced renal function, anemia, atrial fibrillation). Further, patients evaluated as being not eligible for OHT because of their hemodynamic conditions, were not included. Incomplete data was reported for about 75% of patients of the entire cohort. One can assume that in these patients that were excluded from the analysis, PH might be much less frequent.

We chose overall survival as endpoint, which is influenced by a variety of factors, e.g., infection or rejection. The cause of death was available in post-transplant patients, but not standardized assessed. Alternative causes of death might dilute or distort death because of PH and right heart failure. However, the selective analysis of PH vs. no PH patients should have risen the proportion of deaths from right heart failure. However, these limitations reflect real world conditions, making the study clinically relevant. Multicenter study design and investigation of the impact of two recently popularized hemodynamic parameters (PAPi and DPG) on OHT outcomes are substantial strength of our study.

## Conclusion

In the largest European heart transplant database, increased pre-transplant DPG was related to impaired 5-year post-surgical survival. TPG, PVR and PAPi all had poor ability to discriminate survivors from non-survivors. Low PAPi and high PVR were shown to predict graft failure.

## Data availability statement

The original contributions presented in the study are included in the article/Supplementary material, further inquiries can be directed to h.grahn@uke.de.

## Ethics statement

Ethical review and approval was not required for the study on human participants in accordance with the local legislation and institutional requirements. Written informed consent for participation was not required for this study in accordance with the national legislation and the institutional requirements.

## Author contributions

TW, HG, CM, and PK wrote the manuscript draft. TW performed the data analysis. HG and TW designed the study. JS contributed to data analysis and interpretation. HR, AB, and KS contributed to study design and discussion of results. All authors contributed to manuscript writing and read and approved the final manuscript.
