# Supplementary material for: Impact of diastolic pulmonary gradient and pulmonary artery pulse index on outcomes in heart transplant patients—Results from the Eurotransplant database
Source: Front Cardiovasc Med. 2022 Dec 16;9:1036547. doi: 10.3389/fcvm.2022.1036547 (PMC9800977; doi:10.3389/fcvm.2022.1036547)
Supplement: Supplementary file 1 [file Data_Sheet_1.docx]

**Supplementary material**

**Impact of diastolic pulmonary gradient and pulmonary artery pulse index on outcomes in heart transplant patients—Results from the Eurotransplant database**

Wagner T, Magnussen C, Bernhardt A, Smits JM, Steinbach K, Reichenspurner H, Kirchhof P, Grahn H.

**Table S1**

| **Correlation of sPAP and…** | **TPG** |  | **DPG** |  | **Compare of correlations** | |
| --- | --- | --- | --- | --- | --- | --- |
|  | r |  | r |  | *z* | *p* |
| All patients (N = 2149) | 0.470 |  | -0.115 |  | 36.9 | <.001 |
| Only PH (N = 1407) | 0.438 |  | -0.105 |  | 28.8 | <.001 |
| TPG > 15 mmHg (N = 97) | 0.473 |  | -0.170 |  | 7.2 | <.001 |
| PVR > 3 WU (N = 311) | 0.428 |  | -0.146 |  | 13.6 | <.001 |

DPG, diastolic pulmonary artery pressure-to-pulmonary capillary wedge pressure gradient; PH, pulmonary hypertension; PVR, pulmonary vascular resistance; TPG, transpulmonary gradient.

**Table S2**

|  | **TPG** | | **PVR** | |
| --- | --- | --- | --- | --- |
|  | **Median (IQR)** | ***p*** | **Median (IQR)** | ***p*** |
| DPG < 3 mmHg  DPG ≥ 3 mmHg | 7 *(2 – 12)*  12 *(7 – 17)* | <.001 | 2.20 *(0.72 – 3.68)*  3.39 *(1.74 – 5.04)* | <.001 |
| DPG < 5 mmHg  DPG ≥ 5 mmHg | 7 *(2 – 12)*  14 *(8 – 20)* | <.001 | 2.31 *(0.82 – 3.80)*  3.56 *(1.69 – 5.43)* | <.001 |
| DPG < 7 mmHg  DPG ≥ 7 mmHg | 7 *(2 – 12)*  16 *(9 – 23)* | <.001 | 2.38 *(0.86 – 3.90)*  3.71 *(1.78 – 5.64)* | <.001 |
| DPG < 10 mmHg  DPG ≥ 10 mmHg | 8 *(3 – 13)*  20 *(10 – 30)* | <.001 | 2.42 *(0.84 – 4.00)*  3.69 *(1.80 – 5.58)* | <.001 |

*p*-values are calculated by Mann-Whitney *U*-test. DPG, diastolic pulmonary artery pressure-to-pulmonary capillary wedge pressure gradient.

**Table S3**

|  | **mPAP ≥ 25 mmHg and TPG > 12 mmHg** | | **mPAP ≥ 25 mmHg and PVR > 3 WU** | |
| --- | --- | --- | --- | --- |
|  | **Median survival time (days)** | **Log rank *p*** | **Median survival time (days)** | **Log rank *p*** |
| DPG < 3 mmHg  DPG ≥ 3 mmHg | 1811  1204 | 0.030 | 1144  1043 | 0.040 |
| DPG < 5 mmHg  DPG ≥ 5 mmHg | 1556  1204 | 0.404 | 1137  976 | 0.005 |
| DPG < 7 mmHg  DPG ≥ 7 mmHg | 1201  1650 | 0.191 | 1100  1095 | 0.321 |
| DPG < 10 mmHg  DPG ≥ 10 mmHg | 1263  1792 | 0.085 | 1103  930 | 0.112 |

*estimated survival probability never have reached 50%.

DPG, diastolic pulmonary artery pressure-to-pulmonary capillary wedge pressure gradient; mPAP, mean pulmonary artery pressure; TPG, transpulmonary gradient.

**Table S4**

| **Kaplan Meier curves Fig. 2 and 3: Patients at risk** | | | | | | |
| --- | --- | --- | --- | --- | --- | --- |
|  | OHT | 30 days | 12 months | 24 months | 48 months | 60 months |
| DPG <3 mmHg | 1045 | 746 | 610 | 506 | 298 | 202 |
| DPG ≥3 mmHg | 361 | 241 | 180 | 146 | 90 | 60 |
| DPG <5 mmHg | 1199 | 854 | 690 | 572 | 340 | 228 |
| DPG ≥5 mmHg | 207 | 133 | 100 | 80 | 48 | 34 |
| DPG <7 mmHg | 1288 | 912 | 730 | 602 | 357 | 240 |
| DPG ≥7 mmHg | 118 | 76 | 60 | 50 | 31 | 22 |
| DPG <10 mmHg | 1348 | 949 | 759 | 627 | 371 | 249 |
| DPG ≥10 mmHg | 58 | 39 | 31 | 26 | 17 | 13 |
| PAPi ≥1.84 | 678 | 494 | 402 | 326 | 186 | 128 |
| PAPi <1.84 | 603 | 398 | 314 | 260 | 156 | 106 |
| PVR ≥3 WU | 267 | 244 | 178 | 125 | 35 | 12 |
| PVR <3 WU | 505 | 479 | 371 | 245 | 66 | 29 |
| TPG ≥15 mmHg | 255 | 236 | 181 | 143 | 80 | 55 |
| TPG <15 mmHg | 1155 | 1072 | 854 | 660 | 354 | 239 |
